# Acknowledgement of manuscript reviewers 2014

**DOI:** 10.1186/s40199-015-0095-8

**Published:** 2015-02-11

**Authors:** Mohammad Abdollahi, Mohammad Ali Faramarzi, Alireza Foroumadi, Kambiz Gilani, Hossein Khalili, Mahnaz Khanavi, Ali Akbar Moghadamnia, Shekoufeh Nikfar, Ali Nokhodchi, Roja Rahimi, Mohammad Reza Rouini, Alireza Vatanara

**Affiliations:** DARU Journal of Pharmaceutical Sciences, Tehran, Iran

## Abstract

The editors of *DARU Journal of Pharmaceutical Sciences* would like to thank all our reviewers who have contributed to the journal in Volume 22 (2014).

**Akbar Abdollahiasl**

Iran

**Farid Abedin Dorkoosh**

Iran

**Bahareh Abtahi Naeini**

Iran

**Reza Ahmadkhaniha**

Iran

**Sayed Mortaza Alavi Shoushtari**

Iran

**Alireza Aliabadi**

Iran

**Majed Al-Shaeri**

Saudi Arabia

**Amir Amani**

Iran

**Massoud Amanlou**

Iran

**Mohsen Amin**

Iran

**Ghazal Ansari**

Iran

**Amir Ansaripour**

Netherlands

**Kamaran Aryana**

Iran

**Ali Asadipour**

Iran

**Manouchehr Ashrafpour**

Iran

**Abbas Azadmehr**

Iran

**Homa Azizian**

Iran

**Mohammad Babaeian**

Iran

**Zaheer Babar**

New Zealand

**Haji Bahadar**

Pakistan

**Pejman Bakhtiari**

United States of America

**Behzad Baradaran**

Iran

**Ayse Nursen Basaran**

Turkey

**Joaquim Bosch**

Spain

**Jeffrey Brent**

United States of America

**Abdol Majid Cheraghali**

Iran

**Angela Dago**

Spain

**Simin Dashti-Khavidaki**

Iran

**Ilker Dastan**

Turkey

**Siavoush Dastmalchi**

Iran

**Luiza Dias**

Brazil

**Bharat Dixit**

India

**Reza Dowlatabadi**

Iran

**Najmeh Edraki**

Iran

**Saeed Emami**

Iran

**Mohammad Ali Faramarzi**

Iran

**Shadi Farsaei**

Iran

**Gholamhossein Farzandi**

Iran

**Loghman Firoozpour**

Iran

**Alireza Foroumadi**

Iran

**Akbar Fotouhi**

Iran

**Padideh Ghaeli**

Iran

**Alireza Ghaffarieh**

United States of America

**Doaa Ghareeb**

Egypt

**Younes Ghasemi**

Iran

**Kambiz Gilani**

Iran

**Mohammad Hadi Goharbari**

Iran

**Gianpaolo Guzzi**

Italy

**Farzin Hadizadeh**

Iran

**Homa Hajimehdipoor**

Iran

**Javad Hamedi**

Iran

**Mehrdad Hamidi**

Iran

**Hamed Hamishehkar**

Iran

**Hadi Hamishehkar**

Iran

**Shirin Hasani-Ranjbar**

Iran

**Amir Hashemi**

Iran

**Atieh Hashemi**

Iran

**Mohammad Bagher Hashemi**

Iran

**Salman Hassanzadeh**

Sweden

**A. Wallace Hayes**

United States of America

**Marjan Heidary**

Iran

**Bahram Hemmateenejad**

Iran

**Narjes Hendouei**

Iran

**Farshad Homayouni Moghadam**

Iran

**Seyed Jalal Hosseinimehr**

Iran

**Narges Hosseinmardi**

Iran

**Hossein Hosseinzadeh**

Iran

**Takashi Ikejima**

China

**Mona Jaberidoost**

Iran

**Samineh Jafari**

Iran

**Fakhreddin Jamali**

Canada

**Gholamreza Karimi**

Iran

**Iman Karimzadeh**

Iran

**Mehdi Khabazkhoob**

Iran

**Ali Khalaj**

Iran

**Hossein Khalili**

Iran

**Mahnaz Khanavi**

Iran

**Mohsen Khatibi**

Iran

**Payam Khazaeli**

Iran

**Reza Khodarahmi**

Iran

**Mehdi Khoobi**

Iran

**Mehdi Khoshneviszadeh**

Iran

**Maija Konstenius**

Sweden

**Jayashri Kulkarni**

Australia

**Pravir Kumar**

India

**Caterina La Porta**

Italy

**Mohammad Mahdavi**

Iran

**Soleiman Mahjoub**

Iran

**Ghorban Maliji**

Iran

**Azadeh Manayi**

Iran

**William Manzanares**

Uruguay

**Faheem Maqbool**

Pakistan

**Ali Akbar Moghadamnia**

Iran

**Hamidreza Mohammadi**

Iran

**Mostafa Mohammadi**

Iran

**Elham Mohit**

Iran

**Mojtaba Mojtahedzadeh**

Iran

**Aram Mokarizadeh**

Iran

**Dariush Moslemi**

Iran

**Sara Mostafalou**

Iran

**Amrollah Mostafazadeh**

Iran

**Guoying Mu**

Chile

**Hamid Nadri**

Iran

**Nastaran Nafissi-Varcheh**

Iran

**Chanin Nantasenamat**

Thailand

**Latifeh Navidpour**

Iran

**Shekoufeh Nikfar**

Iran

**Ali Nokhodchi**

United Kingdom

**Mohsen Pakzad**

Iran

**Hadi Parsian**

Iran

**Poopak Pir**

Iran

**Morteza Pirali-Hamedani**

Iran

**Mahdi Pouramir**

Iran

**Eslam Pourbasheer**

Iran

**Mohammad Reza Pourmand**

Iran

**Atiar Rahman**

Bangladesh

**Sara Rasoul-Amini**

Iran

**Mohammad Amin Rezvanfar**

Iran

**Amin Rostami-Hodjegan**

United Kingdom

**Daniele Rubert Nogueira**

Brazil

**Mina Saeedi**

Iran

**Soodabeh Saeidnia**

Iran

**Maliheh Safavi**

Iran

**Mahmood Saghaei**

Iran

**Amirhossein Sakhteman**

Iran

**Pooneh Salari**

Iran

**Sajad Salehi**

United States of America

**Satyajit Sarker**

United Kingdom

**Parisa Sarkhail**

Iran

**Habib A. Shojaei Saadi**

Canada

**Fatemeh Soleymani**

Iran

**Kambiz Soltaninejad**

Iran

**Azita Hajhossein Talasaz**

Iran

**Nasser Vahdati-Mashhadian**

Iran

**Ronny Vargas**

Costa Rica

**Alireza Vatanara**

Iran

**Ana Vila Verde**

Germany

**Khushwant S. Yadav**

India

**Che-Ming Yang**

Taiwan

**Mohammad Hossein Yazdi**

Iran

**Fatemeh Yousefbeyk**

Iran

**Ebrahim Zabihi**

Iran

**Bijan Zare**

Iran

**Mohsen Zeeb**

Iran

